# Spousal violence against women and its association with sociodemographic factors and husbands’ controlling behaviour: the findings of Myanmar Demographic and Health Survey (2015–2016)

**DOI:** 10.1080/16549716.2020.1844975

**Published:** 2020-11-20

**Authors:** Tayzar Tun, Per-Olof Ostergren

**Affiliations:** Division of Social Medicine and Global Health, Department of Clinical Sciences, Faculty of Medicine, Lund University, Lund, Sweden

**Keywords:** Intimate partner violence (IPV), marital control, Myanmar-couples, Demographic Health Survey (DHS), policy recommendations

## Abstract

**Background**: Spousal violence is the most common domestic violence against women and a growing public health problem globally. As a behaviour, marital control is commonly accepted as a precursor to spousal violence.

**Objective**: This study examines the prevalence of different types of spousal violence among women in Myanmar and their association with sociodemographic factors and husbands’ controlling behaviour.

**Methods**: This study used data from the Myanmar Demographic and Health Survey (MDHS) 2015–2016. Based on the responses of 3,425 ever-married women, cross-tabulations (Chi-squared test) and multivariate logistic regressions were applied to examine the association between controlling behaviour by husbands and lifetime physical, sexual and emotional spousal violence against Myanmar women. Synergy factor and population attributable fraction were estimated to recommend preventive strategies.

**Results**: The prevalence of lifetime physical violence was 16.8%, of sexual violence 3.8%, of emotional violence 15.9%, and of husband’s controlling behaviour 30.2%. Women who were exposed to controlling behaviour by their husbands reported higher likelihoods of lifetime physical spousal violence (OR = 3.7; 95% CI: 3.0–4.7), lifetime sexual spousal violence (OR = 5.3; 95% CI: 3.3–8.6), and lifetime emotional spousal violence (OR = 5.6; 95% CI: 4.4–7.2). Controlling behaviour by husbands was attributed to 22.0% of lifetime physical spousal violence; and to 24.5% of lifetime sexual spousal violence and to 24.8% of lifetime emotional spousal violence in this sample of Myanmar women. Additional associated factors of spousal violence were poor wealth status, women’s wife-beating justification, exposure to parental violence, and alcohol abuse among husbands.

**Conclusion**: Controlling behaviour by husbands was significantly associated with higher likelihoods of lifetime spousal violence among ever-married Myanmar women in this study. These findings reflect an obvious need for policy development and preventive strategies against marital controlling behaviour in Myanmar.

## Background

Globally, violence against women has been identified as a severe violation of human rights and has also been made a public health priority [1]. Among different forms of violence against women, spousal violence is the most common type all over the world [2]. It occurs in various forms, e.g. physical, sexual and emotional abuse committed by current or former partners within an intimate relationship [3]. According to the global report on violence 2014 by the World Health Organization (WHO), about one in three women in spousal partnerships have experienced partner violence in their lifetime, and the highest proportion was observed in the Southeast Asian region (estimated to 38%) [4].

In several global studies performed by the WHO, it was found that spousal violence is not only associated with adverse health consequences for women such as injuries, physical disabilities, pregnancy-related complications – including unwanted pregnancies, abortions and even death – but also with severe health issues among their children such as a lack of immunization, vulnerability to diarrhoea and other infections and developmental problems [3]. Therefore, spousal violence has a substantial negative impact not only on women but also on the future of the next generation – thus threatening population health and other development aspects of a country.

Myanmar, a middle-income country of 53 million inhabitants [5], is located in the Southeast Asian and also faces the burden of spousal violence. In the first-ever Myanmar Demographic and Health Survey (MDHS) 2015–2016, the prevalence of spousal violence was estimated at 21% among ever-married women aged 15–49 [6]. According to a World Bank report, to date, Myanmar is one of a series of countries that lacks specific legislation against domestic violence as well as spousal violence [7,8]. Additionally, Myanmar also needs to establish a national preventive program on spousal violence.

Moreover, in Myanmar, a limited number of studies on spousal violence have been performed. In one of these studies, spousal violence was found to be associated with factors like having “witnessed parental violence, husbands’ unemployment, husbands’ frequent alcohol use, and women’s ‘feminist attitudes’ [9]. In another study which used the MDHS data (2015–2016) regarding ever-married women in Myanmar, it was found that individual factors such as younger age, lower wealth status and exposure to parental spousal violence were associated with a higher prevalence of physical and/or sexual spousal violence as well as some relationship factors such as ‘marital control behaviour by husband’ and ‘justification of wife beating’ [10]. A qualitative study among married Myanmar women stated that there are inequitable power relations within marriages in Myanmar society. According to this study, this resulted in practices of controlling behaviour by husbands regarding women’s social relations, economic resources and sexual agency; which then led to a high level of spousal violence among married women [11]. These authors suggested that the factor of ‘husband’s controlling behaviour’ should be highlighted in the prevention efforts regarding spousal abuse. Apart from the studies previously mentioned, no other studies about this topic in Myanmar currently exist. Therefore, it is evident that further studies examining spousal violence against Myanmar women and its association with marital controlling behaviour by husbands are needed for supporting necessary legislation and prevention programs against spousal violence throughout the country.

In the perspective of feminist theory, spousal violence is a result of gender inequality, and men use spousal abuse as well as controlling behaviour to dominate women, especially in male-dominated societies [12]. Controlling behaviour within a relationship is defined as a systematic effort of a partner to control the other’s activities, movements and social interactions with outsiders [13]. According to available literature, Myanmar society adheres to a notion that men have an inborn ‘holiness’ [14] and ‘superiority over women’ [15]; resulting into patriarchal communities in which men are primary breadwinners, decision makers and the dominant individual in families as well as in intimate relationships [16]. Therefore, it can be assumed that there would be a strong association between marital controlling behaviour and spousal violence in a traditional Myanmar family. Nevertheless, there is a scarcity of research on male marital controlling behaviour within the context of spousal violence in Myanmar.

It could be noted that the previous nationwide study about spousal violence in Myanmar was mainly focused on physical and sexual abuse [10]. According to MDHS (2015–2016), about 14% of ever-married Myanmar women were estimated to suffer from emotional spousal violence, which was the second most common type of spousal abuse [6]. This reflects that emotional violence should be included in further research.

In conclusion, the aim of this study was to examine the prevalence of different types of spousal violence among women in Myanmar and their association with sociodemographic factors and husbands’ controlling behaviour. To accomplish the aim, this study applied the MDHS data (2015–2016).

## Methods

This study has a cross-sectional design, and utilized data from a nationally representative survey with standardized questionnaires, which was part of a global Demographic and Health Surveys (DHS) program that included a domestic violence module. The used module was adapted to Myanmar culture [6].

### Sample size and study population

Through the two-stage sampling design, 442 clusters were selected first from the 4,000 primary sampling units (PSU) and then 30 households were chosen from each cluster, resulting in a total of 13,260 households participating in the Myanmar Demographic and Health Survey (2015–2016). A random woman in the age-bracket 15–49 from half of the selected households was eligible to be interviewed with domestic module questionnaires in the survey. About 1% of the eligible women were excluded from the survey because of incomplete interviewing process. So, the survey collected information from 4,563 women, of which 3,425 were ever-married women, 15–49 years old and with complete information regarding the variables used in our study. These women make up our study sample.

### Measures

#### Outcome variables

*Lifetime physical spousal violence –* was affirmed if the respondent’s current or former husband(s) committed one of the following: (i) ‘pushing, shaking or throwing something at her; (ii) slapping her; (iii) twisting her arm or pulling her hair; (iv) punching her with his fist or with something that could hurt her; (v) kicking her, dragging her, or beating her up; (vi) trying to choke her or burn her on purpose; (vii) threatening or attacking her with a knife, gun, or any other weapon’ [6].

*Lifetime sexual spousal violence –* was affirmed if the respondent’s current or former husband(s) committed one of the following: (i) ‘physically forcing her to have sexual intercourse with him; (ii) physically forcing her to perform any other sexual acts; (iii) forcing her with threats or in any other way to perform sexual acts’ [6].

*Lifetime emotional spousal violence* – was affirmed if the respondent’s current or former husband(s) committed any one of the following: (i) ‘saying or doing something to humiliate her in front of others; (ii) threatening to hurt or harm her or someone close to her; (iii) insulting her or making her feel bad about herself’ [6].

Each of the outcome variables was scored as 0 (if the respondent did not have experience of any of the quoted events) and 1 (if the respondent had experience of at least one of the quoted events). The coding method was the same for the three types of lifetime spousal violence.

#### Main exposure variable

*Husband’s controlling behaviour* **–** was selected as the main exposure of the study. It referred to any one of following behaviours by current or former husband: (i) ‘being jealous or angry if she talks or talked to other men; (ii) frequently accusing her of being unfaithful; (iii) not permitting her to meet her female friends; (iv) trying to limit her contact with her family; (v) insisting on knowing where she is or was at all time’ [6].

For statistical analyses, husband’s controlling behaviour variable was transformed into a dichotomous variable by coding 0 (if the respondent answered ‘No’ to all response alternatives) and 1 (if the respondent answered ‘Yes’ to any one of the response alternatives).

#### Other exposure variables

Based on the available literature [2,3] and findings of the previous researches about spousal violence in Myanmar [9,10], the following factors were selected as other exposure variables of this study.

*Wealth status*, categorized as low wealth status (combination of poorest and poorer status) and middle or high wealth status (combination of middle, richer and richest status) was selected as a demographic factor variable for this study.

*Respondent’s characteristics* included (i) Respondent’s *Age* – this variable was categorized into 15–29 years and 30–49 years; (ii) *Education*, categorized as none or primary education and secondary or higher education; (iii) *Wife-beating justification* – the respondent was asked whether hitting or beating a wife by a husband is justified in the following situations (a) going out without telling her husband, (b) neglecting her children, (c) arguing with her husband, (d) burning the food, (e) refusing to have sexual intercourse with her husband; and after combing the responses, this variable was transformed into a dichotomous format containing 0 (if the respondent answered ‘no’ to all situations and 1 if the respondent answered ‘yes’ to any one of situations); (iv) *Exposure to parental violence*, was categorized as No or Yes.

*Husband’s characteristics* – (i) *Age*, was categorized as 15–29 years and 30 years and above; (ii) *Education*, was categorized as none or primary education and secondary or higher education; (iii) *Alcohol abuse*, categorized as ‘No’ if the husband had never demonstrated drunkenness and ‘Yes’ if the husband admitted to having been drunk sometimes or often.

### Statistical analysis

All data analyses for this study were performed by the Statistical Package for the Social Sciences (SPSS) version 25 [17]. Firstly, descriptive statistics exploring the baseline characteristics of the study population was applied. After that, cross-tabulations between exposures and each of lifetime physical, sexual and emotional violence were performed together with Pearson’s Chi-squared test (χ^2^) to determine the associations as well as the statistical significance of the difference between exposure variables and outcomes. Logistic regression analyses were conducted to assess the statistically significant associations between exposures and outcomes. The results were reported in odds ratios with 95% confidence intervals. The significance of the associations was statistically accepted if *p*-values of the associations were <0.05.

To assess the possible confounding effects of other exposure variables, the multivariate analytic Model 1 contained husband’s controlling behaviour and demographic factor variable (wealth status). In Model 2, exposure variables that reflect respondent characteristics (age, education, wife-beating justification and exposure to parental violence) were further added to the variables in Model 1. Model 3 included the variables of Model 2, plus variables reflecting husband’s characteristics (age, education and alcohol abuse).

Additionally, to support the formulation of possible intervention strategies, this study also estimated the Synergy Factor (SF) and Population Attributable Fraction (PAF) of the main exposure and other important exposures that showed a statistically significant association with an outcome in the final Model 3.

To compute the Synergy Factor (SF), a new dummy variable for the combination of husband’s controlling behaviour and significant exposure (x) was created by designing four new values such as ‘No control and No x’, ‘No control but x’, ‘Control but No x’ and ‘Both control and x’. After that, the Synergy Factors between husband’s controlling behaviour and significant exposures was calculated by using the following algorithm, which was adapted from previous research about synergy factors [18];

$$SF\;=\;OR_{12}/\left(OR_{1}\times \;OR_{2}\right)$$

where SF = synergy factor between exposure 1 (main exposure) and exposure 2 (other significant exposure in Model 3)

OR_1_ = odds ratio for exposure 1 alone

OR_2_ = odds ratio for exposure 2 alone

OR_12_ = odds ratio for the combined effect of exposure 1 and exposure 2

If SF > 1, a positive interaction or synergy between exposure 1 and exposure 2 is present.

If SF < 1, a negative interaction or antagonism between exposure 1 and exposure 2 is present.

Population Attributable Fraction (PAF) was calculated by using the following algorithm, which was adapted from a previous study that applied odds ratios to compute PAFs [19];

$$PAF\;=\;P\;\times \;\{\left(OR-1\right)\;/\;OR\}$$

where PAF = population attributable fraction of an exposure

P = prevalence of the exposure in the study population

OR = the effect size or the odds ratio of the exposure (in this study, OR = adjusted odds ratio of exposure from the final regression models)

## Results

### Prevalence of lifetime physical, sexual and emotional violence

Among the study population of total 3,425 ever-married Myanmar women, 16.8% had experienced physical violence, 3.8% sexual violence and 15.9% emotional violence in their lifetime (Table 1).

**Table 1.** Baseline characteristics of spousal violence and exposures among ever-married Myanmar women aged 15–49 (*N* = 3425)^a^

Variables	*N*	%
**Forms of spousal violence**		
Physical violence	575	16.8
Sexual violence	131	3.8
Emotional violence	544	15.9
**Main exposure**		
Husband’s controlling behaviour		
No	2379	69.8
Yes	1029	30.2
**Demographic factor**		
Wealth status		
Low	1551	45.3
Middle or high	1874	54.7
**Respondent’s characteristics**		
Age (years) – *M* (*SD*)	34.67 (8.13)	
15–29	988	28.8
30–49	2437	71.2
Education		
None or primary	2180	63.7
Secondary or higher	1244	36.3
Wife-beating justification		
No	1509	49.9
Yes	1515	50.1
Exposure to parental violence		
No	2588	79.2
Yes	678	20.8
Residence		
Urban	832	24.3
Rural	2593	75.7
**Husband’s characteristics**		
Age (years) – *M* (*SD*)	37.45 (9.17)	
15–29	662	21.2
≥30	2468	78.8
Education		
None or primary	1899	56.7
Secondary or higher	1449	43.3
Alcohol abuse		
No	1812	52.9
Yes	1613	47.1

^a^Among 3,425 ever-married women; 225 women married more than once, 143 were widows, 128 were divorced, and 24 were separated from their husbands at the time of the survey.

Data source: Myanmar Demographic and Health Survey (2015–2016).

*N*, number of participants; *M*, mean; SD, standard deviation.

### Husband’s controlling behaviour and selected characteristics

In total, 30.2% of the respondents reported exposure to controlling behaviour by a husband (Table 1). Nearly half (45.3%) of the study population were poor, and the majority (71.2%) were between 30 and 49 years of age. Most of them (75.7%) lived in rural areas. Only 36.3% of the women had a secondary or higher level of education. Half (50.1%) of the participants justified wife-beating by a husband. Twenty per cent of the respondents had a history of exposure to parental violence. The majority (78.8%) of the husbands were 30 years of age or older. Fifty-seven per cent of the husbands had no education or just primary level education. Among all respondents’ husbands, 47% had a history of alcohol abuse.

### Associations between husband’s controlling behaviour, selected characteristics and three types of lifetime spousal violence after multivariate logistic analyses

Husband’s controlling behaviour was significantly associated with lifetime physical violence (OR = 4.2; 95% CI: 3.5–5.1) before adjusting for any potential confounding factors (Table 2). In the final logistic regression Model 3, it was found that the association was reduced, but remained statistically significant (OR = 3.7; 95% CI: 3.0–4.7), even after adjusting for potential confounding factors such as demographic factors, respondent’s and husband’s characteristics. Moreover, in the fully adjusted model (Model 3), it was also found that wealth status, some respondent’s characteristic variables (wife-beating justification and exposure to parental violence) and husband’s characteristic variable (alcohol abuse) were statistically significantly associated with lifetime physical violence (Table 3).

**Table 2.** Crude associations between exposures and lifetime spousal violence among ever-married Myanmar women aged 15–49 (*N* = 3425)

	Lifetime physical violence (*n* = 575)		Lifetime sexual violence (*n* = 131)		Lifetime emotional violence (*n* = 544)	
Variables	% (*n*) with experience	Crude OR (95% CI)	% (*n*) with experience	Crude OR (95% CI)	% (*n*) with experience	Crude OR (95% CI)
**Main exposure**						
Husband’s controlling behaviour						
No	10.1 (241)	1	1.5 (35)	1	8.0 (190)	1
Yes	32.2 (331)	**4.2 (3.5–5.1)**	9.3 (96)	**6.9 (4.7–10.2)**	34.2 (352)	**6.0 (4.9–7.3)**
**Demographic factor**						
Wealth status						
Middle or high	13.2 (247)	1	3.1 (59)	1	13.0 (244)	1
Low	21.1 (328)	**1.8 (1.5–2.1)**	4.6 (72)	**1.5 (1.1–2.1)**	19.3 (300)	**1.6 (1.3–1.9)**
**Respondent’s characteristics**						
Age, years						
30–49	16.3 (398)	1	3.7 (89)	1	15.4 (375)	1
15–29	17.9 (177)	1.1 (0.9–1.4)	4.3 (42)	1.2 (0.8–1.7)	17.1 (169)	1.1 (0.9–1.4)
Education						
Secondary or higher	14.7 (183)	1	3.5 (44)	1	15.0 (187)	1
None or primary	18.0 (392)	**1.3 (1.1–1.5)**	4.0 (87)	1.1 (0.8–1.6)	16.4 (357)	1.1 (0.9–1.3)
Wife-beating justification						
No	14.6 (221)	1	3.0 (45)	1	15.0 (226)	1
Yes	19.5 (295)	**1.4 (1.2–1.7)**	4.8 (72)	**1.6 (1.1–2.4)**	17.7 (268)	**1.2 (1.01–1.5)**
Exposure to parental violence						
No	13.7 (354)	1	3.0 (77)	1	13.1 (339)	1
Yes	27.7 (188)	**2.4 (2.0–3.0)**	6.5 (44)	**2.3 (1.6–3.3)**	25.5 (173)	**2.3 (1.9–2.8)**
**Husband’s characteristics**						
Age, years						
≥30	15.5 (383)	1	3.3 (82)	1	13.7 (337)	1
15–29	14.2 (94)	0.9 (0.7–1.2)	3.3 (22)	1.0 (0.6–1.6)	15.0 (99)	1.1 (0.9–1.4)
Education						
Secondary or higher	15.5 (225)	1	3.0 (43)	1	14.8 (214)	1
None or Primary	17.7 (337)	1.2 (0.98–1.4)	4.5 (85)	**1.5 (1.1–2.2)**	16.9 (320)	1.2 (0.97–1.4)
Alcohol abuse						
No	9.8 (178)	1	2.2 (39)	1	8.4 (153)	1
Yes	24.6 (397)	**3.0 (2.5–3.6)**	5.7 (92)	**2.8 (1.9–4.0)**	24.2 (391)	**3.5 (2.8–4.2)**

Bold notes significant associations with *p*-value <0.05.

Data source: Myanmar Demographic and Health Survey (2015–2016).

OR, odds ratio; CI, confidence interval; *N*, number of participants; *n*, number of cases.

**Table 3.** Adjusted odds ratios (OR with 95% CI) between husband’s controlling behaviour and lifetime spousal violence (*N* = 3425)

		Lifetime physical spousal violence			Lifetime sexual spousal violence			Lifetime emotional spousal violence		
		Model 1: adjusted for demographic factor	Model 2: Model 1 + respondent’s characteristics	Model 3: Model 2 + husband’s characteristics	Model 1: adjusted for demographic factor	Model 2: Model 1 + respondent’s characteristics	Model 3: Model 2 + husband’s characteristics	Model 1: adjusted for demographic factor	Model 2: Model 1 + respondent’s characteristics	Model 3: Model 2 + husband’s characteristics
Husband’s controlling behaviour	No vs. Yes	4.3(3.6–5.2)*	4.4 (3.5–5.4)*	3.7 (3.0–4.7)*	6.9(4.7–10.3)*	6.7(4.3–10.4)*	5.3 (3.3–8.6)*	6.1 (5.0–7.4)*	6.1 (4.9–7.6)*	5.6 (4.4–7.2)*
**Demographic factor**	**Categories**									
Wealth status	Middle or high vs. low	1.8(1.5–2.2)*	1.7 (1.4–2.2)*	1.6 (1.2–2.0)*	1.5(1.1–2.2)**	1.5(1.02–2.3)**	1.2 (0.8–1.9)	1.7 (1.4–2.1)*	1.7 (1.4–2.1)*	1.7 (1.3–2.2)*
**Respondent’s characteristics**	**Categories**									
Age	30–49 vs. 15–29		0.9 (0.8–1.2)	1.2 (0.9–1.6)		0.9 (0.6–1.3)	1.1 (0.6–2.0)		1.0 (0.8–1.3)	0.9 (0.6–1.2)
Education	Secondary or higher vs. None or primary		1.3(1.02–1.6)**	1.2(0.9–1.6)		1.1 (0.7–1.7)	1.1 (0.7–1.9)		1.2 (0.9–1.5)	1.1 (0.8–1.4)
Wife-beating justification	No vs. Yes		1.3(1.01–1.5)**	1.3(1.01–1.6)**		1.5 (0.99–2.3)	1.6 (0.98–2.5)		1.1(0.9–1.4)	1.1 (0.9–1.4)
Exposure to parental violence	No vs. Yes		2.0 (1.6–2.6)*	2.0 (1.6–2.6)*		1.8(1.2–2.8)**	1.6 (0.99–2.6)		1.9(1.5–2.4)*	1.8 (1.4–2.4)*
**Husband’s characteristics**	**Categories**									
Age	30–49 vs. 15–29			0.7 (0.5–1.04)			0.8 (0.4–1.7)			1.3 (0.9–2.0)
Education	Secondary or higher vs. None or primary			1.0 (0.8–1.3)			1.6 (0.95–2.7)			1.2 (0.9–1.5)
Alcohol abuse	No vs. Yes			2.6 (2.1–3.3)*			2.1 (1.3–3.3)**			3.2 (2.5–4.1)*

Data source: Myanmar Demographic and Health Survey (2015–2016).

*p-value < 0.001, **p-value < 0.05.

OR, odds ratio; CI, confidence interval; *N*, number of participants.

Without adjustment for any confounding factors, the association between lifetime sexual violence and husband’s controlling behaviour was found to be statistically significant (OR = 6.9; 95% CI: 4.7–10.2) (Table 2). After controlling for all potential confounding factors (Model 3), the effect estimate of the association between husband’s controlling behaviour and lifetime sexual violence decreased slightly; however, the association was still statistically significant (OR = 5.3; 95% CI: 3.3–8.6). In addition to the main exposure, it was found that a husband’s characteristic variable (alcohol abuse) was statistically significantly associated with lifetime sexual violence in the fully adjusted model (Model 3) (Table 3).

Regarding lifetime emotional violence, its association with husband’s controlling behaviour was statistically significant (OR = 6.0; 95% CI: 4.9–7.3) before adjusting for any potential confounder (Table 2). In the fully adjusted model (Model 3), a marginal decline was found regarding the effect estimate of the association between husband’s controlling behaviour and lifetime emotional violence, but the association remained statistically significant (OR = 5.6; 95% CI: 4.4–7.2). In the fully adjusted model, statistically significant associations were also found between lifetime emotional violence and wealth status, respondent’s exposure to parental violence, and husband’s alcohol abuse (Table 3).

### Synergy factors

Five out of the totaleight synergy factors were very close to 1, which means that there was no synergy. The remaining SFs ranged between 1.2 and 0.9, expressing marginal evidence of synergy or antagonism. In summary, the synergy analyses showed general additive effects rather than synergistic ones between husband’s controlling behaviour and other significant exposures (Table 4).

**Table 4.** Synergy factors (SF) concerning the effects on spousal violence among ever-married Myanmar women aged 15–49 (*N* = 3425)

	Lifetime physical violence (*n* = 575)		Lifetime sexual violence (*n* = 131)		Lifetime Emotional violence (*n* = 544)	
Variables	Crude OR (95% CI)	*SF*	Crude OR (95% CI)	*SF*	Crude OR (95% CI)	*SF*
**Husband’s controlling behaviour and wealth status**						
No control and not poor	1		-		1	
No control but poor	**1.8 (1.4–2.4)**		-		**1.7 (1.2–2.2)**	
Control but not poor	**4.3 (3.3–5.7)**		-		**6.0 (4.5–7.9)**	
Control and poor	**7.9 (6.0–10.4)**	1.0	-	-	**10.3 (7.7–13.8)**	1.0
**Husband’s controlling behaviour and wife-beating justification**^a^						
No control and 0	1		-		-	
No control and 1–5	1.2 (0.9–1.6)		-		-	
Control and 0	**3.8 (2.8–5.1)**		-		-	
Control and 1–5	**5.4 (4.1–7.1)**	1.2	-	-	-	-
**Husband’s controlling behaviour and exposure to parental violence**						
No control and no exposure	1		-		1	
No control but exposed	**2.2 (1.6–3.0)**		-		**2.1 (1.5–3.0)**	
Control but no exposure	**4.1 (3.2–5.2)**		-		**6.0 (4.7–7.6)**	
Both control and exposure	**9.1 (6.8–12.3)**	1.0	-	-	**12.0 (8.9–16.4)**	1.0
**Husband’s controlling behaviour and husband’s alcohol abuse**						
No control and no alcohol abuse	1		1		1	
No control but alcohol abuse	**2.9 (2.2–3.8)**		**2.2 (1.1–4.4)**		**3.2 (2.3–4.3)**	
Control but no alcohol abuse	**4.1 (3.0–5.7)**		**6.1 (3.1–12.0)**		**5.5 (3.9–7.7)**	
Both control and alcohol abuse	**11.0 (8.3–14.5)**	0.9	**14.4 (7.9–26.2)**	1.1	**18.3 (13.5–25.0)**	1.0

Bold notes a statistically significant odds ratio with *p*-value <0.05.

^a^Wife-beating justification was statistically associated with lifetime physical violence only, in the final models.

Data source: Myanmar Demographic and Health Survey (2015–2016).

OR, odds ratio; CI, confidence interval; SF, synergy factor; *N*, number of participants; *n*, number of cases.

### Population Attributable Fractions (PAF) between exposure and three types of lifetime spousal violence

We found that 22% of lifetime physical violence, 24.5% of lifetime sexual violence and 24.8% of lifetime emotional violence could be linked to controlling behaviour by husbands. Alcohol abuse by husbands was attributed to 29% of lifetime physical violence, 24.7% of lifetime sexual violence and 32.4% of lifetime emotional violence (Table 5).

**Table 5.** Percentage of population attributable fraction (PAF) of significant exposures among ever-married Myanmar women aged 15–49 (*N* = 3425)

				Percentage of population attributable fraction (PAF)		
Exposures	Prevalence of exposure within the study population (P) (%)	Outcome	OR (95% CI)	Point estimate	Lower-limit point estimate	Upper-limit point estimate
Husband’s controlling behaviour	30.2	Physical violence	3.7 (3.0–4.7)	**22.0**	20.1	23.8
		Sexual violence	5.3 (3.3–8.6)	**24.5**	21.0	26.7
		Emotional violence	5.6 (4.4–7.2)	**24.8**	23.3	26.0
Wealth status (poor)	45.3	Physical violence	1.6 (1.2–2.0)	**17.0**	7.6	22.7
		Emotional violence	1.7 (1.3–2.2)	**18.7**	10.5	24.7
Wife-beating justification	50.1	Physical violence	1.3 (1.01–1.6)	**11.6**	0.5	18.8
Exposure to parental violence	20.8	Physical violence	2.0 (1.6–2.6)	**10.4**	7.8	12.8
		Emotional violence	1.8 (1.4–2.4)	**9.2**	5.9	12.1
Husband’s alcohol abuse	47.1	Physical violence	2.6 (2.1–3.3)	**29.0**	24.7	32.8
		Sexual violence	2.1 (1.3–3.3)	**24.7**	10.9	32.8
		Emotional violence	3.2 (2.5–4.1)	**32.4**	28.3	35.6

Data source: Myanmar Demographic and Health Survey (2015–2016).

OR, adjusted odds ratio in the final model; CI, confidence interval. *N*, number of participants.

## Discussion

In this representative sample of ever-married Myanmar women, 17% had experienced lifetime physical violence; 4% lifetime sexual violence, and 16% lifetime emotional violence. Thirty per cent of the study population was exposed to controlling behaviour by husbands and we found that this factor was strongly associated with lifetime spousal violence. Controlling behaviour by husbands was attributed to 22% of lifetime physical violence and 25% of lifetime sexual violence as well as emotional violence in our sample. Moreover, some highly prevalent associated factors such as poverty, husband’s alcohol abuse, justification of wife-beating and exposure to parental violence were also associated with lifetime spousal violence among ever-married Myanmar women.

Compared to some regional countries and WHO’s multi-countries study, the prevalence of lifetime physical spousal violence in Myanmar (16.8%) – was higher than urban Japan (13%) but lower than Bangladesh (48.7%) and provincial Peru (61%) [20,21]. Lifetime sexual spousal violence in Myanmar (3.8%) was lower than in other countries in the region – urban Japan (6%), Laos (7.2%), and then in provincial Ethiopia (59%) [21,22]. Regarding lifetime emotional spousal violence, Myanmar had a higher prevalence (15.9%) than India (13.8%) but lower than Vietnam (54%) and provincial Ethiopia (75.1%) [21,23,24]. The prevalence of husband’s controlling behaviour was 30.2% in Myanmar, which was higher than in urban Japan (21%) however, lower than in urban Tanzania (90%) [21]. In summary, Myanmar women seem to have been exposed to a lower burden of lifetime spousal violence and controlling behaviour by their husbands than many of the world’s countries. However, it should be considered that some variations in collection methods, response rates and questionnaires could affect the comparability of results [4].

The fully adjusted analysis in our study showed that controlling behaviour by husbands was associated with a four-fold higher likelihood of physical spousal violence, a five-fold higher likelihood of sexual spousal violence and a sixfold higher likelihood of emotional spousal violence among ever-married Myanmar women in their lifetime. This supports the hypothesis that all types of spousal violence (physical, sexual and emotional) against ever-married women are associated with controlling behaviour by husbands in Myanmar. This finding is in line with a feminist perspective of spousal violence [12,25,26] – claiming that husbands commonly use controlling behaviour and spousal abuse to dominate women. The results were also in line with previous studies from Thailand and Nepal [27,28] as well as other national representative studies from Nigeria and Turkey [29,30] which found that more marital control exerted by husbands raised the likelihood of experience with spousal violence among their wives.

According to evidence from studies in England and the USA [31,32], emotional abuse and marital controlling behaviour are risk factors for physical and sexual spousal violence in couples. In our sample, lifetime emotional violence was the likeliest form of violence, but we also note a potential burden of physical and sexual violence among women in Myanmar where marital controlling behaviour by husbands is generally high.

Corresponding with results from other studies in Asia and Pacific regional countries [33–36], our study also found that poor wealth status was associated with lifetime physical and emotional violence among ever-married Myanmar women. This supports the hypothesis that spousal violence can be a result of poverty-related stress, or a functional tool to overcome the pressure of men having the role as the primary provider for their families. This pressure may be even more so in poor households [37]. Additionally, poverty-related stress can also limit the ability of a woman to abandon an abusive intimate partner relationship [29,38], which leads to physical and emotional spousal violence for prolonged periods of a woman’s life. Since about 26% of the country’s population live in poverty [39], Myanmar needs to focus on this factor when tackling the burden of spousal violence against women.

Similarly, women’s exposure to parental violence was found to be strongly associated with lifetime physical and emotional violence in Myanmar. This is in accordance with the results from studies from neighbouring countries [36,40]. This point is important in preventing spousal violence among the younger population because Myanmar does not have enough social welfare facilities to address this issue.

This study also found that wife-beating justification was a significantly associated factor for lifetime physical violence. This finding is in line with the results of the WHO’s multi-country study [41], and a similar Chinese study [42]. In Bangladesh [43] and Vietnam [44], it was found that women who live in patriarchal societies tend to accept men’s supremacy over women as well as the husband’s right to beat his wife as a punitive act. As such, the relation between wife-beating justification and lifetime physical violence in our study may be a result of patriarchal norms in Myanmar society.

Alcohol abuse by husbands increased the likelihood of lifetime physical, sexual and emotional violence among the women of this study. This is in agreement with previous studies from China, Congo, and Spain, which found that the greater the alcohol abuse by husbands the higher the likelihood of spousal violence against women [42,45,46]. As a result, Myanmar also needs to mitigate alcohol abuse by men in order to reduce spousal violence.

This study also found that there was no obvious synergistic effect between controlling behaviour by husbands and other significant associated factors such as poor wealth status, women’s wife-beating justification, exposure to parental violence, and alcohol abuse by husbands in Myanmar. This finding can be helpful for formulating preventive strategies, especially in a resource-limited setting. It might not be necessary to implement simultaneous interventions of two associated factors at a time.

Moreover, the Population Attributable Fractions (PAFs) estimated by this study will be useful for prioritizing preventive strategies in Myanmar. The mitigation of poverty, controlling behaviour by husbands, and alcohol abuse in households has a theoretical potential of reducing the burden of lifetime physical spousal violence and lifetime emotional spousal violence among ever-married Myanmar women. However, the burden of lifetime sexual spousal violence among ever-married Myanmar women is predicted to be effectively reduced only by implementing strategies mitigating marital controlling behaviour and alcohol abuse in husbands. This reflects that poverty reduction alone could not prevent the occurrence of sexual spousal violence in Myanmar.

According to our study, Myanmar husbands practise marital controlling behaviour to dominate their wives as a traditional norm of male superiority, or to overcome the poverty-related stress. If husbands abuse alcohol, and their wives have experienced parental violence as well as justifying wife-beating norms, the risk for spousal violence was increased. To reduce the burden of spousal violence in Myanmar, gender roles have to be changed towards a more equitable direction – i.e. increasing the empowerment of women, and promoting non-patriarchal norms in the community.

### Strengths and limitations

A main strength of the present study is that it uses the data set of the Myanmar Demographic and Health Survey (2015–2016), which is representative of country’s population and has a high response rate [6]. We therefore believe that the findings of this study can be generalised to ever-married women in the entire population of Myanmar. It also entails that the study used well-validated measures for all background, main exposure, and outcome variables – which also allowed for adequate control for important confounders. Since the sample size was large, it was also possible to test some tentative synergistic relations between exposure variables.

This study also has some limitations. Firstly, this study applied a cross-sectional study design; therefore, causality between exposure and outcome variables cannot be conclusively assessed. Another limitation of this kind of study, is that cultural factors could be important for the propensity of reporting some of the phenomena under investigation, which calls for some caution when making comparisons of prevalence across countries and cultures. However, it may be assumed that the associations between the used variables represent a fairly general pattern, because it seems to be very similar between different cultural settings.

Thirdly, this study probably has the risk of misclassifications. For example, poor women could systematically underreport the occurrence of spousal violence regardless of their exposure to controlling behaviour by their husbands, which could lead to differential misclassification and an underestimation of the strength of some of the associations [47].

Fourthly, since this study was a secondary data analysis on the MDHS 2015–2016, it was not possible to examine some risk factors of spousal violence, which were not collected in the survey. As a result, the results of this study could not exclude the existence of residual confounding [47].

Fifthly, instead of Relative Risks (RR), the use of odds ratios (OR) in the calculation of population attributable fractions (PAFs) is likely to yield some overestimation since certain exposures displayed a high prevalence in the study population [47].

## Conclusion

This study verified the high burden of spousal violence among ever-married Myanmar women. It also revealed a high prevalence of marital controlling behaviour and its strong association with lifetime physical, sexual and emotional spousal violence in Myanmar. Furthermore, it also demonstrated the contributions of wealth status, women’s wife-beating justification, exposure to parental violence, and alcohol abuse by husbands to lifetime spousal violence in Myanmar families. Regarding policy recommendations, the health-care sector and social welfare sector should educate the community about the impact of spousal violence, and encourage the reporting of marital controlling behaviour. Early notification and prompt mitigating action against marital controlling behaviour can prevent the occurrence of spousal violence among women. Meanwhile, poverty reduction programs should be reinforced, especially in households of the lowest socioeconomic status. The economic empowerment of vulnerable communities can facilitate the impact of other factors such as the transformation of culture and gender norms related to spousal violence. Additionally, stricter regulation of alcohol use could also reduce the burden of spousal violence in Myanmar. To reduce the psychological trauma as well as the likelihood of accepting spousal violence norms among those who have witnessed parental spousal violence, counselling services should be integrated into the current health-care programs of Myanmar.

## Paper context

The recent Myanmar Demographic and Health Survey (2015-2016) found that 21% of ever-married Myanmar women suffered from spousal violence. However, Myanmar still lacks specific legislations and preventive programs against gender-based violence. This study revealed the correlation of intimate partner violence with some of the main socio-cultural factors, especially husband’s controlling behaviour. The results of this study are unique and yield supportive recommendations regarding the future implementation of preventive strategies against gender-based violence in Myanmar.
